# Role of Food Insecurity in Outbreak of Anthrax Infections among Humans and Hippopotamuses Living in a Game Reserve Area, Rural Zambia

**DOI:** 10.3201/eid2309.161597

**Published:** 2017-09

**Authors:** Mark W. Lehman, Allen S. Craig, Constantine Malama, Muzala Kapina-Kany’anga, Philip Malenga, Fanny Munsaka, Sergio Muwowo, Sean Shadomy, Melissa A. Marx

**Affiliations:** Centers for Disease Control and Prevention, Atlanta, Georgia, USA (M.W. Lehman, S. Shadomy);; Centers for Disease Control and Prevention, Lusaka, Zambia (A.S. Craig, C. Malama, M.A. Marx);; Ministry of Health, Lusaka, Zambia (M. Kapina-Kany’anga, P. Malenga);; Provincial Health Office, Chipata, Zambia (F. Munsaka);; District Health Office, Chama, Zambia (S. Muwowo)

**Keywords:** anthrax, Bacillus anthracis, one health, hippopotamus, food safety, food insecurity, zoonoses, Chama, Zambia, bacteria

## Abstract

In September 2011, a total of 511 human cases of anthrax (*Bacillus anthracis*) infection and 5 deaths were reported in a game management area in the district of Chama, Zambia, near where 85 hippopotamuses (*Hippopotamus amphibious*) had recently died of suspected anthrax. The human infections generally responded to antibiotics. To clarify transmission, we conducted a cross-sectional, interviewer-administered household survey in villages where human anthrax cases and hippopotamus deaths were reported. Among 284 respondents, 84% ate hippopotamus meat before the outbreak. Eating, carrying, and preparing meat were associated with anthrax infection. Despite the risk, 23% of respondents reported they would eat meat from hippopotamuses found dead again because of food shortage (73%), lack of meat (12%), hunger (7%), and protein shortage (5%). Chronic food insecurity can lead to consumption of unsafe foods, leaving communities susceptible to zoonotic infection. Interagency cooperation is necessary to prevent outbreaks by addressing the root cause of exposure, such as food insecurity.

During August–September 2011, a total of 85 hippopotamuses (*Hippopotamus amphibious*) died of suspected anthrax (*Bacillus anthracis*) infection in a game management area along the South Luangwa River near the district of Chama in northeastern Zambia (Figure 1) (*1*). At least 521 suspected human anthrax cases and 5 deaths were reported near this area during this period. Residents of the area near the river had reportedly found dead hippopotamuses and subsequently butchered, cooked, and consumed meat from the dead animals, which was thought to be the cause of the human outbreak. As previously reported, most human cases of anthrax infection were cutaneous infections, with most patients initially having papular lesions (95%) and the rest having lymphadenopathy and gastrointestinal symptoms (*1*). Most cases resolved after the patient received a course of oral ciprofloxacin. Some additional animal species were reported by local wildlife staff to have been affected, but no empiric data were found to corroborate those reports.

**Figure 1:**
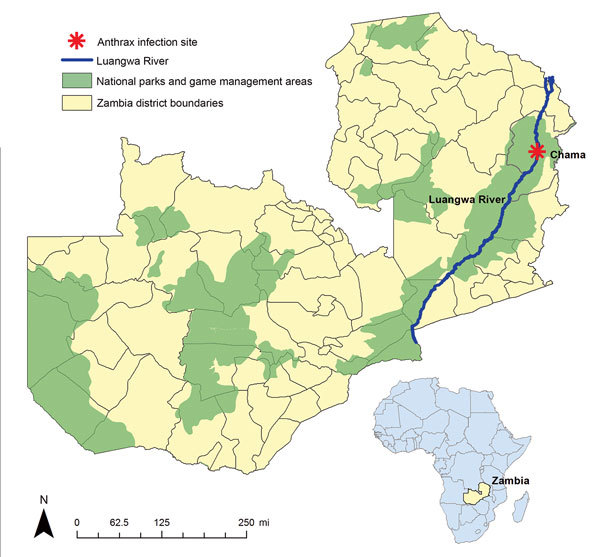
Location of an anthrax outbreak that originated in a game management area along the South Luangwa River in the Chama District of northeastern Zambia, 2011. Inset map shows location of Zambia in Africa.

Anthrax outbreaks associated with animals are common and reported worldwide. Herbivores are thought to have onset of disease after ingesting spores in soil, water, or on vegetation. Reports of anthrax outbreaks occurring in wild and domestic animals in Africa have usually been associated with the dry season and have stopped with the onset of the rainy season (*2*,*3*). Outbreaks can begin with wildlife, expand into domestic livestock, and ultimately affect humans (*4*,*5*). When anthrax outbreaks occur in national parks in Africa and are limited to the wildlife, the outbreak is usually allowed to run its natural course without any human intervention. Multiple challenges make it impractical to vaccinate free-ranging wildlife populations; sometimes vaccine programs are initiated but usually only to protect endangered species or populations at high risk (*6*–*8*).

Anthrax outbreaks associated with hippopotamuses have been reported previously in Zambia, Uganda, Zimbabwe, and South Africa (*3*,*9*–*11*). Human cases associated with wildlife outbreaks in Africa are generally not well-documented but are known to occur (*5*).

Zambia’s Chama District (population 103,894) borders Malawi, in what is currently known as Muchinga Province (Figure 1) (*12*). Nearly 93% of the district’s residents live in rural areas, and overall population density is 5.9 persons/km^2^ (*12*). The rural population resides in small villages largely accessible only by all-terrain vehicles. Communication in many parts of the district is only possible through 2-way radios. Chama is within a game management area that includes the South Luangwa River and contains rich flora and fauna, including hippopotamuses but also other foragers and predators. Because of Chama’s status as a game management area, residents are not permitted to protect crops from foragers or hunt on area grounds, which are overseen by the Zambia Wildlife Authority (ZAWA). Food and water are scarce for animals and humans during Zambia’s dry season, generally May–November. A delay in the annual rainy season, usually December–March, can put farmers at risk for low crop production, as was the case in 2011 (*13*). During this period, animals forage deep into riverbeds in search of water and food, digging up and activating dormant anthrax spores. Residents, for whom food can also be scarce under these conditions, have been known to consume animals they find dead in their area (Figure 2).

**Figure 2:**
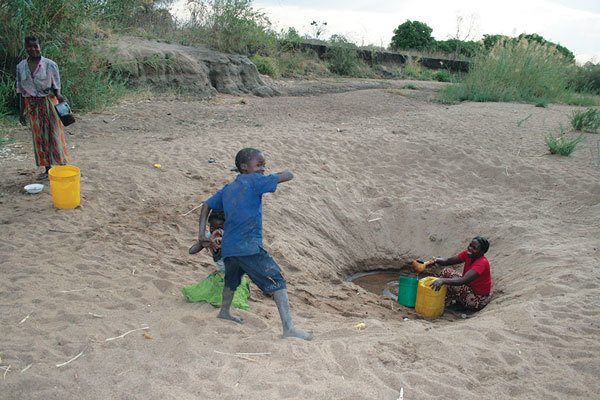
A family searching for water by digging deep into a dried riverbed during the dry season in northeastern Zambia.

In September 2011, a few months after the first human anthrax cases were reported in the district, a team of epidemiologists, health officers, and environmental health technicians from the Zambia Central Ministry of Health, the Eastern Provincial Health Office, and Chama District Health Office conducted a preliminary investigation of the possible outbreak under their jurisdiction. Shortly thereafter, upon formal request, epidemiologists from the US Centers for Disease Control and Prevention joined the Zambia team to conduct this outbreak investigation in an effort to further inform prevention activities.

Specifically, we aimed to shorten the outbreak by identifying and eliminating any remaining exposures and by recommending mitigation and prevention strategies for this and future outbreaks. We also wanted to determine the riskiest exposures so that educational messages could be properly tailored. Here we report the results of a household survey used to identify the most common exposures associated with human anthrax and to determine how food insecurity contributed to consumption of the anthrax-contaminated meat and anthrax infection among residents of this game management area.

## Methods

We conducted a cross-sectional, interviewer-administered household survey in 3 villages with access to riverbeds where hippopotamuses died: Chikwa, Chigoma, and Chimpamaba. These villages’ estimated combined population of adults >15 years of age is 6,553 (*12*). A questionnaire (Technical Appendix) was developed in English and translated into Senga, the local language spoken in these areas. The questionnaire was designed to complement and expand on the data initially collected in the district by local public health officials. It captured age, sex, and occupation and yes/no responses to several questions about symptoms, exposure to hippopotamuses, food sources, and whether respondents would eat meat from an animal they found dead. Those who responded that they would eat animals they found dead were asked an open-ended question to elucidate their reasons for doing so. Interviewers and supervisors were trained on procedures for conducting the survey before fieldwork commenced. Interviewers and supervisors tested questions on each other, which resulted in improvements to question wording. They also practiced administering the survey on one another before interviewing community participants.

Three teams administered the questionnaire, each team made up of a trained District Health Office staff supervisor and 6 trained volunteer interviewers. Each team was assigned to 1 of the 3 communities. Interviewers selected every fifth household they encountered in each village for inclusion and interviewed all adults >15 years of age living in selected households. No incentives were offered for participation. Interviewers read each prospective participant a description of the survey, highlighting that it was voluntary and could be stopped at any time by request. The Zambia Ministry of Health deemed this outbreak investigation to be exempt from Research Ethics Committee review. The investigation protocol was reviewed by the CDC-Zambia office and CDC’s National Center for Emerging and Zoonotic Infectious Diseases, in accordance with institutional review policies. The protocol was determined to be nonresearch under 45 CFR 46 §102(d) and therefore did not require Institutional Review Board review.

Data from completed questionnaires were entered into an Access database (Microsoft, Redmond, WA, USA) and analyzed by using Epi Info version 3.5.1 (CDC, Atlanta, GA, USA). We defined a case as illness in a respondent who reported having had anthrax infection diagnosed by a healthcare worker since July 2011 and compared demographic characteristics and risk factors by case status by using χ^2^ and *t* tests. Associations were quantified with simple and multivariable logistic regression.

**Figure 3:**
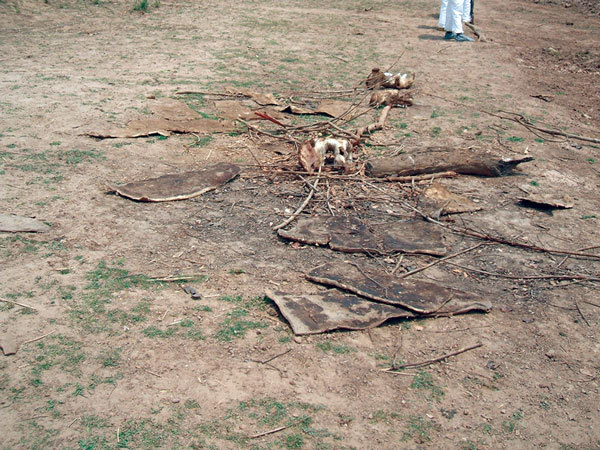
Hippopotamus bones and hides left behind after butchering of animals that were found dead on a river bank and later identified as the source of anthrax causing an outbreak among humans in northeastern Zambia, 2011.

**Figure 4:**
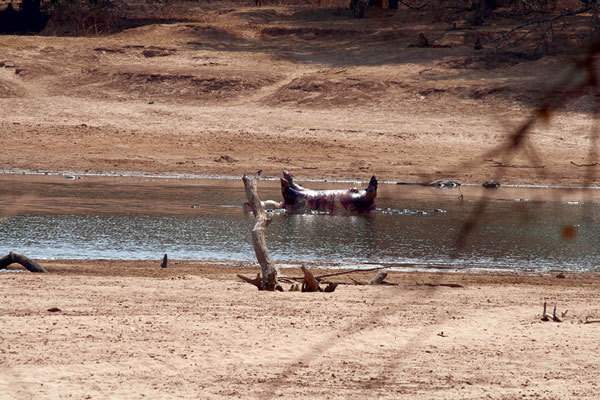
A dead hippopotamus floating down the South Luangwa River in northeastern Zambia during an anthrax outbreak in 2011.

The second part of the investigation involved going into the field to view 3 of the areas where most of the infected hippopotamuses were found. The site visits occurred within about a month after the outbreak and were conducted to better understand the topography and the range of the animals and to identify any additional animal species affected by anthrax. All sites were areas frequented by the exposed human populations and were <1 km from residents’ homes. The site surveys were not exhaustive; they focused on areas with known human-animal interaction (Figure 3) and recently discovered dead hippopotamuses (Figure 4). N95 masks, Tyvek suits, and rubber boots were worn during site visits when members exited vehicles. We used these observations and the results of the household-level surveys to develop recommendations to mitigate and control future spread of the infection to humans, animals, and the environment.

## Results

All 284 household members (≈4% of the population of the villages) in the 87 households selected agreed to be interviewed (mean 3 participants per household). In total, 31 (11%) of participants reported having anthrax infection diagnosed by a healthcare worker since July 2011; another 137 (48%) reported not having anthrax infection diagnosed, and 116 (41%) did not know whether they had anthrax infection diagnosed. We assumed that those who did not know did not have anthrax infection diagnosed. Male respondents accounted for 48% (n = 136) of total participants but 68% (n = 21) of those reporting having anthrax infection diagnosed, compared with 36% of cases occurring among female respondents (p<0.001) (Table 1). Of the 96 persons who answered the occupation-related question, 91 (95%) listed their occupation as farmer. This finding was not surprising given the rural setting of the outbreak. Because the responses for occupation were so homogenous, occupation was not evaluated as a risk factor. The median age of persons having received an anthrax diagnosis since July 2011 was 33 years, similar to the median age of all participants (32 years) (Table 1). The most common signs and symptoms reported by those reporting having been diagnosed with anthrax included myalgia, skin lesions, fatigue, diarrhea, and fever (Table 2).

**Table 1:** Demographic characteristics of respondents to a survey conducted after an outbreak of anthrax infections among humans and hippopotamuses living in a game reserve area, by case status, Chama District, Zambia, September 2011*

Characteristic	Persons with anthrax diagnosed since July 2011, n = 31	Persons without anthrax diagnosed since July 2011, n = 137
Median age (range), y	33 (15–72)	34 (15–83)
Sex, %		
M	68	36
F	32	64

*116 (41%) of survey participants reported that they did not know whether they had had anthrax diagnosed since July 2011; of these, median age was 30 (range 15–77) years, and 52% were male.

**Table 2:** Signs and symptoms of respondents reporting having had anthrax in survey conducted after outbreak of anthrax infections among humans and hippopotamuses living in a game reserve area, Chama District, Zambia, September 2011*

Signs/symptoms	No. (%) respondents
Myalgia	21 (67)
Skin lesion	18 (58)
Fatigue	18 (58)
Diarrhea	17 (54)
Fever	16 (52)

*Anthrax infection diagnosed since July 2011.

Most participants (238 [84%]) reported having eaten hippopotamus meat at the time of the outbreak. Participants who ate the meat were 9 times (95% CI 1.3–369.3 times) more likely to report having had anthrax than those who did not eat the meat. Carrying hippopotamus meat (odds ratio 5.3, 95% CI 2.0–15.4) and preparing it for cooking (odds ratio 3.3, 95% CI 1.1–13.7) were also significantly associated with anthrax infection. After controlling for having eaten the hippopotamus meat, 3 activities (skinning, carrying, and cutting the meat) were all still significantly associated with reported anthrax infection (Table 3).

**Table 3:** Association of anthrax diagnosis with specific activities involving hippopotamus carcasses based on responses to a survey conducted after an outbreak of anthrax infections among humans and hippopotamuses living in a game reserve area, Chama District, Zambia, September 2011*

Activity	No. (%) persons		OR (95% CI)	aOR (95% CI)
	With anthrax diagnosed since July 2011, n = 31	Without anthrax diagnosed, n = 137		
Skinning	14 (45)	8 (6)	13.3 (4.4–41.5)	12.0 (4.3–36.5)
Cutting	28 (90)	70 (51)	8.9 (2.5–47.5)	8.1 (2.2–29.2)
Eating	30 (97)	106 (77)	8.8 (1.3–369.3)	–
Carrying	24 (77)	54 (39)	5.3 (2.0–15.4)	4.4 (1.7–11.8)
Preparing	27 (87)	92 (67)	3.3 (1.1–13.7)	2.1 (0.5–11.8)
Cooking	27(87)	93(68)	3.2 (1.0–13.2)	2.0 (0.5–1.1)
Drying	21(68)	64(47)	2.4 (1.0–6.1)	1.7 (0.6–4.5)

*aOR, adjusted odds ratio (adjusted for eating hippopotamus meat); OR, odds ratio.

Most people surveyed (216 [76%]) reported they would not eat meat from a dead hippopotamus knowing now that it can cause anthrax infection, but 65 (23%) of all respondents and 5 (16%) of the 31 respondents in whom cases were reported said that they would eat meat from a dead hippopotamus despite this knowledge. Of the 65 participants saying they would eat the meat if given the chance again, reasons given were because they lacked other options for a side dish (“relish”) to Nshima, the maize-based staple food (44 [73%]); lacked meat (14 [22%]); suffered from hunger (4 [7%]); or lacked protein (3 [5%]), in addition to other less commonly reported reasons (Table 4).

**Table 4:** Reasons for intending to eat meat again from hippopotamuses suspected to have died from anthrax among 65 persons who reported consuming dead hippopotamus meat in a survey conducted after an outbreak of anthrax infections among humans and hippopotamuses living in a game reserve area, Chama District, Zambia, September 2011

Reason	No. (%) respondents*
Lack of side dish	44 (73)
Lack of meat	14 (22)
Hunger	4 (7)
Lack of protein	3 (5)

*Respondents could provide >1 response.

The investigation team also visited several field sites. At 1 of the sites, previous human interaction with dead hippopotamuses was evident. Bones and hides were strewn across a large area. Evidence of multiple campfires were found in the vicinity of the hippopotamus remains (Figure 3). According to a Zambia Ministry of Health official and others on the investigation team who had visited the site earlier, the strewn animal parts appeared to have been from the initial human contact with the dead hippopotamuses. Residents appeared to stop handling dead hippopotamuses after human anthrax cases were detected and linked to contact with hippopotamus carcasses.

## Discussion

A large outbreak of cutaneous anthrax among humans in the Chama District of Zambia was associated with physical contact with meat from hippopotamuses that had died of anthrax, specifically skinning, carrying, or cutting the meat. Food insecurity was thought to have been the major factor driving the local population to consume meat from dead animals.

Large outbreaks affecting hippopotamus herds occurred within the Luangwa River Valley during 1987–1988 (*7*). Although no human infections were reported in relation to these outbreaks, positive results with low antibody titers against *B. anthracis* were obtained during a 1989 follow-up study from half of the subjects in a small sample of unvaccinated healthy volunteers from Luangwa River Valley villages, suggesting previous exposures in those persons through handling or consumption of meat from anthrax-infected animal carcasses (*7*).

Our findings are subject to a few limitations. In the household surveys, we used self-report of anthrax diagnosis to define a case; however, 41% of participants indicated they did not know whether they had received an anthrax diagnosis. We assumed that these respondents might not have understood the question and had probably not had anthrax diagnosed. Although this assumption is a limitation, it would probably bias our associations toward the null. We also had to use self-report of diagnosis rather than laboratory confirmation. However, separately some hippopotamus and human samples were confirmed in the laboratory as positive for *B. anthracis*, which does strengthen the epidemiologic linkage (*14*). Finally, slight discrepancies can be noted in the number of cases and hippopotamus deaths in this and the 2 other reports describing other aspects of the outbreak and response that have been published; specifically, the numbers of human cases vary from 511 to 521, and the numbers of hippopotamus deaths vary from 81 to 85 (*14*,*15*). This discrepancy likely illustrates the difficulty in describing events in very remote areas.

From this investigation we found that the greatest risk for having anthrax diagnosed came from carrying, skinning, or butchering hippopotamuses. This finding is consistent with other anthrax outbreaks associated with contaminated meat (*11*,*16*).

Recommendations from this investigation built on the initial response of the Zambia Ministry of Health and included community education, enhanced surveillance in human and animal populations, and resolution of food insecurities by working with governmental and nongovernmental agencies. The message to not eat meat from animals found dead was communicated at the time of the initial investigation. On the basis of survey responses indicating persons were no longer touching the meat and that carcasses were no longer being butchered, we think the messages were received and understood by the communities affected. Because the handling of the carcasses proved to be the most important risk factor for anthrax infection, future education campaigns should also focus on avoiding handling animals that have died of unknown causes. In rural Zimbabwe communities where anthrax awareness was high (71.5%) in a 2013 survey, 41% of persons surveyed reported “forgetting about anthrax,” a major reason for consuming meat from anthrax-infected animals (*17*). Residents should be reminded by community-based awareness campaigns or other means of the hazards of consuming meat from animals that have died of unknown causes (*15*).

To inform planning, wildlife authorities should identify high-risk periods and locations for naturally occurring animal outbreaks through ecologic studies that identify conditions favoring anthrax infection among animal populations (*18*–*20*). Wildlife and public health authorities should work together to ensure that community-based campaigns proactively prepare communities for possible outbreaks according to their risk profile (*20*). Community-based interventions should involve residents in addressing communitywide food insecurity and in educating neighbors on the hazards of consuming meat from animals that die of unknown causes (*16*). These interventions should begin before the dry season in outbreak-prone areas.

Questionnaire responses showed that food insecurities appear to be the primary reason for handling and consuming meat from animals found dead. Other countries in Africa have undertaken successful programs to distribute meat from trophy animals to feed communities with limited access to protein while also reducing poaching by local communities (*21*). Such an approach might be considered as a component of a multisectoral solution to address food insecurity and consumption of unsafe foods in Zambia.

Overall, food insecurity throughout sub-Saharan Africa has improved throughout recent years; however, hunger and malnutrition continue to be concerns in many sub-Saharan countries including Zambia. Zambia maintains a food reserve of maize, and it was suggested throughout the investigation that officials should provide additional corn meal as a possible solution for the food shortage. However, populations at risk for food insecurity need better access to balanced diets rather than more carbohydrates (*22*). Our survey respondents highlighted the desire for more fresh fruits and vegetables, which suggests more balanced diets would be welcomed.

Most of the crops grown in this region were cotton and other nonedible, exportable crops. Assistance is needed to help the population better balance subsistence farming with cash crops on small family farms to improve the overall diversity of crops and ultimately mitigate the risk for food insecurity (*22*).

Our household survey aimed to determine the main risk factors for anthrax transmission and the underlying factors driving those infected to risk exposure. Our results suggest the need to address long-standing political and economic issues related to food insecurity in protected areas, as well as an urgent need for better coordination between wildlife management and public health authorities. A more proactive approach could help prevent future outbreaks.

Technical Appendix English version of an investigation form used to interview persons potentially exposed to anthrax during in outbreak in northeastern Zambia, 2011.
