# Supplementary material for: Role of Food Insecurity in Outbreak of Anthrax Infections among Humans and Hippopotamuses Living in a Game Reserve Area, Rural Zambia
Source: Emerg Infect Dis. 2017 Sep;23(9):1471–7. doi: 10.3201/eid2309.161597 (PMC5572886; doi:10.3201/eid2309.161597)
Supplement: Technical Appendix — English version of an investigation form used to interview persons potentially exposed to anthrax during in outbreak in northeastern Zambia, 2011. [file 16-1597-Techapp-s1.pdf]

Article DOI: https://doi.org/10.3201/eid2309.161597

Role of Food Insecurity in Outbreak of Anthrax Infections among Humans and Hippopotamuses Living in a Game Reserve Area, Rural Zambia

Technical Appendix

Chama District Anthrax Exposure Follow-Up Investigation Form

SUPPLEMENTAL MATERIAL

FORM NUMBER:_________ Household number_______

Name of interviewer (First Last)_____________________

DATE OF INTERVIEW:________-________-_________

(dd month yy)

Name of village____

Thank you for agreeing to take part in this survey. This survey is voluntary and you may choose not to answer some or all of it. We are here to gather more information about an illness that may have occurred in this area.

DEMOGRAPHIC INFORMATION

1. SEX: _☐ Male _☐ Female
2. AGE (years)_________________
3. EMPLOYED: _☐ Yes _☐ No
4. IF YES, OCCUPATION:____

RISK EXPOSURES

5. Did you do any of the following activities with **dead hippos** between July 2011 and September 2011?

Skinning _☐ Yes _☐ No

Drying _☐ Yes _☐ No

Cutting meat _☐ Yes _☐ No

Carrying meat _☐ Yes _☐ No

Preparing meat for cooking _☐ Yes _☐ No

Cooking meat _☐ Yes _☐ No

Eating meat _☐ Yes _☐ No

6. If you ate **hippo meat** how was it prepared?

Raw _☐ Yes _☐ No

Roasted _☐ Yes _☐ No

Dried _☐ Yes _☐ No

Boiled _☐ Yes _☐ No

7. Do you have any hippo meat in your house? _☐ Yes _☐ No

8. Did you see any dead hippos in the last week? _☐ Yes _☐ No

9. If yes, where did you see the dead hippos? ____

10. If yes, did you fish in the area the same day as seeing the dead hippo? _☐ Yes _☐ No

11. When did you eat or have contact with **dead hippos**?

Earliest date____-_____-___ Most recent date ___-__________-____

dd month yy dd month yy

12. Did you eat any other game meat between July 2011 and September 2011? _☐ Yes _☐ No

13. Did you eat fish between July 2011 and September 2011? _☐ Yes _☐ No

If you ate fish how was it prepared?

Raw _☐ Yes _☐ No

Roasted _☐ Yes _☐ No

Fried _☐ Yes _☐ No

Dried _☐ Yes _☐ No

Boiled _☐ Yes _☐ No

PAST MEDICAL HISTORY

14. Have you had a skin lesion, similar to the photo shown here, before July 2011? _☐ Yes _☐ No

15. If yes, was it more than one year ago? _☐ Yes _☐ No

16. Have you been diagnosed with anthrax before July 2011? _☐ Yes _☐ No

17. If yes, was it more than one year ago? _☐ Yes _☐ No

18. Do you have any on-going health conditions? _☐ Yes _☐ No _☐ Refused

19. May I ask what your conditions are? _☐ Yes _☐ No

First Condition: ____

Second Condition: ____

Third Condition: ____

CLINICAL INFORMATION

20. Were you ill between July 2011 and September 2011? _☐ Yes _☐ No

(if no skip to **FOOD SECURITY**)

21. Chief Complaint?____

22. Date of onset: ___-________-____ (show calendar to help with date)

dd month yy

23. Did you have any of the following between July 2011 and September 2011?

Cough _☐ Yes _☐ No

If yes, sputum production _☐ Yes _☐ No

If yes, any blood _☐ Yes _☐ No

Shortness of breath _☐ Yes _☐ No

Conjunctivitis _☐ Yes _☐ No

Tender or enlarged lymph nodes _☐ Yes _☐ No

Fever _☐ Yes _☐ No

Headache _☐ Yes _☐ No

Stiff neck _☐ Yes _☐ No

Muscle aches _☐ Yes _☐ No

Fatigue _☐ Yes _☐ No

Joint pains _☐ Yes _☐ No

Confusion _☐ Yes _☐ No

Sore throat _☐ Yes _☐ No

Diarrhea _☐ Yes _☐ No If yes, bloody? _☐ Yes _☐ No

Vomiting _☐ Yes _☐ No If yes, bloody? _☐ Yes _☐ No

Skin lesion(s) _☐ Yes _☐ No

Boil _☐ Yes _☐ No

Black scab (show photo) _☐ Yes _☐ No

24. Where was the black scab?

Face _☐ Yes _☐ No

Was there a cut or scratch here before the lesion? _☐ Yes _☐ No

Neck _☐ Yes _☐ No

Was there a cut or scratch here before the lesion? _☐ Yes _☐ No

Back _☐ Yes _☐ No

Was there a cut or scratch here before the lesion? _☐ Yes _☐ No

Chest _☐ Yes _☐ No

Was there a cut or scratch here before the lesion? _☐ Yes _☐ No

Arm _☐ Yes _☐ No

Was there a cut or scratch here before the lesion? _☐ Yes _☐ No

Hand _☐ Yes _☐ No

Was there a cut or scratch here before the lesion? _☐ Yes _☐ No

Leg _☐ Yes _☐ No

Was there a cut or scratch here before the lesion? _☐ Yes _☐ No

Foot _☐ Yes _☐ No

Was there a cut or scratch here before the lesion? _☐ Yes _☐ No

25. Did you go to a clinical or hospital for care? _☐ Yes _☐ No

26. Name of hospital or clinic____

27. Date you sought care ____-___________-______

dd month yy

28. Were you told you had anthrax? _☐ Yes _☐ No

29. Were you given medication as treatment? _☐ Yes _☐ No

FOOD SECURITY: We understand that access to food in this area is difficult. We would like to ask a few questions to better understand the problem with limited food sources.

30. Do you hunt? _☐ Yes _☐ No

31. Do you farm? _☐ Yes _☐ No

    If yes what crops?

    Maize _☐ Yes _☐ No

    Cotton _☐ Yes _☐ No

    Rice _☐ Yes _☐ No

    Other ___________________________________

32. What is your primary source of food?

    Maize _☐ Yes _☐ No

    Beans _☐ Yes _☐ No

    Game meat _☐ Yes _☐ No

    Ground nuts _☐ Yes _☐ No

    Fish _☐ Yes _☐ No

33. What food would you prefer to substitute for game meat? ____________________________

34. Would you eat meat from an animal you found dead? _☐ Yes _☐ No

35. If yes, why would you eat meat from an animal you found dead?_______________________
